# Prevalence and risk factors for delayed antenatal care visits in Rwanda: an analysis of secondary data from Rwanda demographic health survey 2019-2020

**DOI:** 10.11604/pamj.2023.44.74.37570

**Published:** 2023-02-07

**Authors:** Annet Mulungi, Judith Mukamurigo, Samuel Rwunganira, Kato Njunwa, Joseph Ntaganira

**Affiliations:** 1 Field Epidemiology Training Program, Department of Epidemiology and Biostatistics, School of Public Health, College of Medicine and Health Sciences, University of Rwanda, Kigali, Rwanda,; 2 Department of Epidemiology and Biostatistics, School of Public Health, College of Medicine and Health Sciences, University of Rwanda, Kigali, Rwanda,; 3 Rwanda Biomedical Centre, Ministry of Health, Kigali, Rwanda,; 4 Research, School of Health Sciences, College of Medicine and Health Sciences, University of Rwanda, Kigali, Rwanda

**Keywords:** Antenatal care, delayed, demographic health survey, Rwanda, factors

## Abstract

**Introduction:**

antenatal care (ANC) delivers services to prevent pregnancy complications and provides counseling for birth, and emergency preparedness. Having ANC on time has life-saving potential for the child and mother. Despite improvements in health infrastructure, human resources, and health insurance, hindrances to early ANC visits still exist in Rwanda. This study aimed to investigate the burden and factors associated with delayed ANC visits in Rwanda so that policymakers can develop strategies to promote early ANC visits.

**Methods:**

this is a cross-sectional study using Rwanda demographic health survey (RDHS) 2019-2020 that included 6,039 women that had had a pregnancy in the 5 years preceding the survey. Descriptive analysis was used to determine the prevalence and a multivariable logistic regression model using manual backward stepwise regression was used to identify risk factors for delayed ANC in Rwanda. STATA 16 statistical software was used for all the analyses.

**Results:**

the prevalence of delayed ANC in Rwanda was 41% and the risk factors include: the number of children 4-6 (AOR = 1.4, 95% CI: 1.2-1.6) and 7 or more children (AOR = 1.5, 95% CI: 1.5-2.1) versus less than 3 children, unwanted pregnancy (AOR = 1.7, 95% CI: 1.5-2.0), not covered by health insurance (AOR = 14, 95% CI: 1.2-1.6), woman´s education level: no education (AOR 2.6, 95% CI: 1.6-4.1), primary education (AOR 2.5, 95% CI: 1.6-3.7), secondary education (AOR 2.2, 95% CI: 1.5-3.2), woman´s occupation: informal (AOR 2.3 95% CI: 1.5-3.7) and unemployment (AOR 2.3. 95% CI: 1.4-3.7). **Conclusion:** based on the findings from our study, family planning services should be made available to all women of childbearing age to prevent unwanted pregnancies; female education should be considered a priority, promotion of health insurance coverage and community-based education about reproductive health to encourage the early seeking of care among women of childbearing age.

## Introduction

Antenatal care (ANC) service provides an opportunity to prevent pregnancy-related complications, deliver the counseling for birth, and prepare for possible emergencies [1]. Focused ANC purposes mainly to help women sustain normal pregnancies by timely identifying preexisting conditions and complications during childbirth and promoting well-being [2].

In 2017, it was estimated that 810 women die per day around the world from preventable complications related to pregnancy or childbirth [3]. Sub-Saharan Africa has the highest maternal mortality burden, totaling approximately 66% (196,000) of the estimated worldwide number of maternal deaths in 2017 [4]. The highest neonatal mortality rate of 41% of neonatal deaths worldwide is also observed in sub-Saharan Africa [5]. Some countries in Africa have a lifetime risk of pregnancy or childbirth-related deaths estimated at one in 23 women, compared to one in 7000 women in Northern Europe [6]. Rwanda had a lifetime risk of maternal death of one in 94 in the year 2017, while the proportion of deaths due to maternal causes among women of reproductive age (PM point estimate) was 12% [7].

Having the appropriate ANC on time has life-saving potential for the child and mother [8]. The decreased rates of preterm birth, a common cause of neonatal morbidity and mortality in low-resource settings, have been linked to increased ANC attendance [9]. In 2016, the World Health Organization (WHO) recommended at least 8 ANC visits, with the first visit to be undertaken before the 12^th^ week of pregnancy [8]. Despite growing awareness, delays in ANC persist in sub-Saharan Africa (SSA), and the delays still persist grouped as delays in seeking, reaching, and receiving ANC [10]. Several predictors of delayed initiation of ANC have been identified in low-resource countries and these include: lower education level, low household income, higher cost of care, unemployment [11], pregnancy intention [1], parity [12], exposure to mass media [13], distance from the health facility, health insurance [14] and residence [15].

Rwanda is one of the few countries globally and the only one in the region, that achieved Millennium Development Goals (MDGs) 4 and 5 for reductions in under 5 mortality and maternal mortality [16]. This success is a result of improved health system strengthening through cross-sector collaborations, community-based care, evidence-based policymaking, strong partnership between local and central governments and a high-level political commitment [17]. Rwanda made increasing attendance at ANC a national priority, considering the established facts of the benefits to mothers and neonates [18]. A community health worker (CHW) outreach program to promote ANC attendance was implemented in Rwanda since 2007. This involves community health workers identifying pregnant women, providing prenatal health education, and encouraging attendance at ANC [19].

Although 99% of pregnant women received ANC service, the number of visits was below the standard set by WHO and the Rwanda Ministry of Health, 56% made their first visit before the fourth month of pregnancy compared to 38% in 2010 [18]. Hindrances to early ANC visits still exist despite improvements in health infrastructure, human resources, and health insurance. Therefore, this study aims to investigate the burden and factors associated with delayed ANC visits in Rwanda so that policymakers can develop strategies to promote early ANC visits.

## Methods

**Study setting:** Rwanda is a low-income, landlocked country with an estimated 11 million people living in five regions, with an average of 4.4 persons per household and a gross domestic product per capita of US dollars 816 [4]. The Rwandan health system consists of four levels including referral hospitals, district hospitals, health centers, and health posts. The basic antenatal care services are offered at health centers and health posts and are free of charge while other ANC services are offered at the district and referral hospitals [20]. The community health workers identify pregnant women and refer them to health facilities for ANC services.

**Study design:** this was a cross-sectional study on 6,029 women who attended ANC in Rwanda using secondary data analysis of the 2019-2020 Rwanda Demographic Health Survey (RDHS). The RDHS 2019-2020 is the sixth DHS to be conducted in Rwanda, of which data were collected from November 9^th^, 2019 to July 20^th^, 2020. Data collection was interrupted for 2 months from March 21^st^ to June 7^th^ 2020 because of a nationwide lockdown due to the COVID-19 pandemic. The RDHS 2019-2020 was a cross-sectional survey that was conducted using multistage cluster sampling of villages and households, with stratification of all 30 districts. The study included all women 15-49 years of age from 13,000 households, who had a pregnancy in the last 5 years preceding the survey. The survey respondents answered questions about their reproductive health history, access to health services, recent pregnancy experiences, household assets, and reproductive health practices. If there was more than one pregnancy, the outcomes and predictors were based on their last pregnancy.

**Variable:** the primary outcome variable for this study is delayed ANC, defined as 'No´ ANC visit or having the first ANC visit in the second or third trimester of pregnancy.

**Quantitative variables:** through literature review and data in the DHS, 14 potential factors associated with delayed ANC visits were identified including age in years, number of children, place of residence, place of ANC, marital status, pregnancy (wanted or unwanted), woman´s education, problem with distance to health facility, covered by health insurance, wealth quintile, husband´s education, woman´s employment status, partner´s employment status, knowledge of menstrual cycle.

**Study participants/study size:** all 6,039 women aged 15-49 years who had a pregnancy in the 5 years preceding the RDHS 2019-2020 and included in the dataset were considered for this study.

**Inclusion criteria:** all women aged 15-49 years with complete data on the outcome variable, who had a pregnancy in the 5 years preceding the survey.

**Exclusion criteria:** women aged 15-49 years who had a pregnancy in the 5 years preceding the survey with incomplete data on the outcome variable will be excluded.

**Data source:** this was an analysis done using secondary data of women aged 15-49 from the Rwanda Demographic and Health Survey 2019-2020 [21]. This cross-sectional survey is conducted nationwide including rural and urban areas in all regions.

**Statistical methods:** descriptive analysis in mean with standard deviation, percentages, and frequencies was used to describe the socio-demographic and obstetric characteristics of the study population. Bivariate logistic regression at a 95% confidence interval was used to test for the significance of the association between the socio-demographic characteristics, and obstetric characteristics with delayed ANC visits (dependent variable). All factors with p<0.05 in the design-based chi-square for categorical variables were considered statistically significant for the multivariable logistic regression analysis. All variables were assessed for collinearity and no strong collinearity (>r.0.8) was identified for all covariates. The manual backward stepwise regression was used to come up with the final multivariable logistic regression model of the risk factors for delayed ANC in Rwanda. Significant variables were retained for the final model. Adjusted odds ratio (AOR) and 95% confidence interval (CI) were used to report the magnitude of association between sociodemographic and obstetric characteristics with delayed ANC. Sampling weights and adjustment for clustering and stratification of observations were applied to all analyses. STATA version 16 was used, with svyset commands to apply inverse probability weights that account for oversampling of urban primary sampling units and to adjust for clustering of observations within primary sampling units (PSUs) and stratification by districts. Sampling weights were applied to produce proper representation.

**Ethical considerations:** this study was approved by the Institutional Review Board (IRB) at the University of Rwanda with ethical clearance number 286/CMHS IRB/2022 and authorized to access data on the DHS database. No informed consent was used because it is secondary data, but a confidentiality agreement was signed.

After obtaining approval, de-identified data were downloaded to ensure confidentiality and anonymity. The results of this study will be shared with the Maternal and Child Health Division at Rwanda Biomedical Center, the School of Public Health at the University of Rwanda, or submitted for publication.

## Results

**Participants:** a total of 6,039 respondents were recruited for this study based on the data from the Rwanda demographic health survey 2019-2020.

**Descriptive data:** forty-one percent of women had delayed ANC visits. The mean age of women was 29.1 years, range 15-49, and a standard deviation of 9.9. Table 1 and Table 2 show the sociodemographic and obstetric characteristics of the study participants.

**Table 1:** socio-demographic characteristics of women with delayed ANC in Rwanda

Sociodemographic characteristics	First trimester (timely ANC)	Second or third trimester (delayed ANC)
	**% (95% CI)**	**% (95% CI)**
**Age group n=6,160**		
15-24 (n=1,071)	56.9 (53.8-59.9)	43.1 (40.1,46.2)
25-34 (n=2,932)	63.1 (61.0-65.2)	36.9 (34.8-39)
35-44 (n=1,997)	57.9 (55.1-60.7)	42.1 (39.3-44.9)
45-49 (n=160)	49.9 (41.6-58.2)	50.1 (41.8-58.40
**Marital status (n=5,536)**		
In union (n=5,000)	61.7 (59.9-63.6)	38.3 (36.4-40.1)
Not in union (n=536)	59.0 (53.9-64.0)	41 (36.0-46.1)
**Type of place of residence (n=6,160)**		
Urban (n=1,099)	61.1 (57.2-64.8)	38.9 (35.2-42.8)
Rural (n=5,061)	59.8 (57.9-61.6)	40.2 (38.4-42.1)
**Wealth index combined (n=6,160)**		
Poorest (n=1,387)	53.2 (n=50.2-56.1)	46.8 (43.9-49.8)
Poorer (n=1,183)	56.4 (53.1-59.6)	43.6 (40.4-46.9)
Middle (n=1,201)	61 (57.6-64.3)	39 (35.7-42.4)
Richer (n=1,217)	63.0 (59.6-66.3)	37 (33.7-40.4)
Richest (n=1,172)	67.7 (64.3-70.9)	32.3 (29.1-35.7)
**Husband/partner's education level (n=5,000)**		
No education (n=649)	54.2 (50.3-58.0)	45.8 (42.0-49.7)
Primary (n=3,338)	59.7 (57.5-61.8)	40.3 (38.2-42.5)
Secondary (n=731)	69.0 (65.0-72.7)	31 (27.3-35.0)
Higher (n=281)	84.9 (79.3-89.1)	15.1 (10.9-20.7)
**Woman's education level (n=6,160)**		
No education (n=661)	53.4 (48.9-57.8)	46.6 (42.2,51.1)
Primary (n=3,979)	57.9 (56.1-59.8)	42.1 (40.2-43.9)
Secondary (n=1,244)	64.4 (61.3-67.4)	35.6 (32.6-38.7)
Higher (n=274)	86.3 (81.6-89.9)	13.7 (10.1-18.4)
**Husband/partner's occupation (n=5,000)**		
Formal (n=310)	79.4 (73.7-84.2)	20.6 (15.8-26.3)
Informal (n=4,334)	60.5 (58.6-62.4)	39.5 (37.6-41.4)
Not working (n=355)	61.1 (55.2-66.6)	38.9 (33.4-44.8)
**Woman's occupation (n=6,160)**		
Formal (n=213)	87.7 (82.4-91.5)	12.3 (8.5-17.6)
Informal (n=4,896)	58.7 (56.9-60.4)	41.3 (39.6-43.1)
Not working (n=1,051)	60.7 (56.7-64.6)	39.3 (35.4-43.3)
**Covered by health insurance (n=6,160)**		
No (n=1,106)	50.1 (46.8-53.5)	49.9 (46.5-53.2)
Yes (n=5,054)	62.2 (60.4-63.9)	37.8 (36.1-39.6)
**Distance to health facility (n=6,160)**		
Big problem (n=1,426)	56 (52.9-59.0)	44 (41.0-47.1)
Not a big problem (n=4,733)	61.2 (59.5-63)	38.8 (37.0-40.5)
**Place of ANC visit (n=6,156)**		
Health post/dispensary (n=140)	55.4 (43.5-66.7)	44.6 (33.3-56.5)
Health center (n=5,649)	59.4 (57.7-61.0)	40.6 (39.0-42.3)
District/Provincial hospital (n=174)	62.7 (54.8-69.9)	37.3 (30.1-45.2)
Private or other (n=59)	69 (55.1-80.1)	31 (19.9-44.9)
Referral hospital (n=133)	84.5 (76.9-89.9)	15.5 (10.1-23.1)

ANC: antenatal care

**Table 2:** obstetric characteristics and delayed ANC in Rwanda

Characteristics	First trimester	Second or third trimester
	**(Timely ANC)**	**(Delayed ANC)**
	**% (95% CI)**	**% (95% CI)**
**Total children ever born**		
Less than 3 (n=4,046)	63.9 (62-65.7)	36.1 (34.3-38)
4-6 (n=1,657)	53.9 (51.1-56.8)	46.1 (43.2-48.9)
7 and above (n=456)	47.9 (42.8-53)	52.1 (47.0-57.2)
Total (n=6,160)	60 (58.3-61.6)	40 (38.4-41.7)
**Wanted pregnancy**		
Yes (n=3,600)	66.8 (64.8-68.8)	33.2 (31.2-35.2)
No (n=2,560)	50.4 (48.2-52.6)	49.6 (47.4-51.8)
Total (n=6,160)	60 (58.3-61.6	40 (38.4-41.7)
**Knowledge of ovulatory cycle**		
No (n=4,995)	58.6 (56.8-60.4)	41.4 (39.6-43.2)
Yes (n=1,117)	65.9 (62.6-69.1)	34.1 (30.9-37.4)
Total (n=6,113)	60 (58.3-61.6)	40 (38.4-41.7)

**Outcome data:** Table 3 and Table 4 show the bivariate analysis between socio-demographic, and obstetric factors and delayed ANC. The factors that were statistically significant, (p<0.05), include: age group 25-34 (p<0.001), wealth index poorest (p<0.001) and poorer (p<0.001), husband/partner´s education; no education (p<0.001), primary (p<0.001) and secondary (p<0.001), woman´s education level; no education (p<0.001), primary (p<0.001), secondary (p<0.001), husband/partner´s occupation; informal (p<0.001), not working (p<0.001), woman´s occupation; formal (p<0.001) and not working (p<0.001), not covered by health insurance (p<0.001), distance to health facility being a big problem (p<0.001), place of ANC referral hospital (p<0.001), total number of children 4-6 (p<0.001), 7 and above (p<0.001), unwanted pregnancy (p< 0.001), no knowledge of ovulatory cycle (p<0.001).

**Table 3:** bivariate analysis between sociodemographic characteristics and delayed ANC in Rwanda

Characteristics	COR	95% CI	P-value
**Sociodemographic**			
**Age group**			
15-24 (n=1,071)	Reference		
25-34 (n=2,932)	0.8	0.6-0.9	<0.001
35-44 (n=1,997)	0.9	0.8-1.1	0.614
45-49 (n=160)	1.3	0.9-1.9	0.122
**Marital status**			
In union (n=5,000)	Reference		
Not in union (n=536)	1.1	0.9-1.4	0.32
**Residence**			
Urban (n=1,099)	Reference		
Rural (n=5,061)	1	0.9-1.2	0.556
**Wealth index combined**			
Poorest (n=1,387)	1.8	1.5-2.2	<0.001
Poorer (n=1,183)	1.6	1.3-1.9	<0.001
Middle (n=1,201)	1.3	1.1-1.6	0.003
Richer (n=1,217)	1.2	1.0-1.5	0.041
Richest (n=1,172)	Reference		
**Husband/partner’s education level**			
No education (n=649)	4.7	3.1-7.1	<0.001
Primary (n=3,338)	3.8	2.5-5.6	<0.001
Secondary (n=731)	2.5	1.6-3.8	<0.001
Higher (n=281)	Reference		
**Woman’s education level**			
No education (n=661)	5.5	3.7-8.2	<0.001
Primary (n=3,979)	4.6	3.2-6.5	<0.001
Secondary (n=1,244)	3.4	2.4-5.0	<0.001
Higher (n=274)	Reference		
**Husband/partner's occupation**			
Formal (n=310)	Reference		
Informal (n=4,334)	2.5	1.8-3.5	<0.001
Not working (n=355)	2.5	1.6-3.7	<0.001
**Woman’s occupation**			
Formal (n=213)	Reference		
Informal (n=4,896)	5	3.3-7.6	<0.001
Not working (n=1,051)	4.6	2.9-7.2	<0.001
**Covered by health insurance**			
No (n=1,106)	1.6	1.4-1.9	<0.001
Yes (n=5,054)	Reference		
**Distance to health facility**			
Big problem (n=1,426)	1.2	1.1-1.4	0.001
Not a big problem (n=4,733)	Reference		
**Place of ANC visit**			
Health post/dispensary (n=140)	1.2	0.7-1.9	0.506
Health center (n=5,649)	Reference		
District/Provincial hospital (n=174)	0.9	0.6-1.2	0.409
Private or other (n=59)	0.6	0.3-1.2	0.169
Referral hospital (n=133)	0.2	0.1-0.4	<0.001

ANC: antenatal care; COR: crude odd ratio; CI: confidence interval

**Table 4:** bivariate analysis between obstetric characteristics and delayed ANC in Rwanda of study participants

Obstetric characteristics	COR	95% CI	P-value
**Total children ever born**			
Less than 3 (n=4,046)	Reference		
4-6 (n=1,657)	1.5	1.3-1.7	<0.001
7 and above (n=456)	1.9	1.5-2.3	<0.001
**Wanted pregnancy**			
Yes (n=3,600)	Reference		
No (n=2,560)	2	1.7-2.2	<0.001
**Know ovulatory cycle**			
No (n=4,995)	1.4	1.2-1.5	<0.001
Yes (n=1,117)	Reference		

COR: crude odd ratio; CI: confidence interval

**Main results:** Table 5 shows the multivariable logistic regression model with odds ratio, p-value and confidence intervals for the risk factors of delayed first ANC visit in Rwanda; age group 25-34 (AOR=0.8, 95% CI: 0.6-0.9) and 35-44 (AOR-0.7, 95% CI: 0.6-0.9), number of children 4-6 (AOR=1.4, 95% CI: 1.2-1.6) and 7 or more children (AOR=1.5, 95% CI: 1.5-2.1) versus less than 3 children, unwanted pregnancy (AOR=1.7, 95% CI: 1.5-2.0), not covered by health insurance (AOR=1.4, 95% CI: 1.2-1.6)), Woman´s education; no education(AOR=2.6, 95% CI: 1.6-4.1), primary level (AOR=2.5, 95% CI: 1.6-3.7), secondary level (AOR=2.2, 95% CI: 1.5-3.2) versus higher education level, woman´s occupation; informal (AOR=2.3, 95% CI: 1.5-3.7), not working (AOR=2.3, 95% CI: 1.4-3.7) versus formal employment.

**Table 5:** multivariable logistic regression analysis of factors associated with delayed ANC in Rwanda

Factors	AOR	95% CI	P-value
**Age group**			
15-24 (n=1,071)	Reference		
25-34 (n=2,932)	0.8	0.6-0.9	0.006
35-44 (n=1,997)	0.7	0.6-0.9	0.007
45-49 (n=160)	0.8	0.5-1.2	0.436
**Woman’s education level**			
No education (n=661)	2.6	1.6-4.1	<0.001
Primary (n=3,979)	2.5	1.6-3.7	<0.001
Secondary (n=1,244)	2.2	1.5-3.2	<0.001
Higher (n=274)	Reference		
**Woman’s occupation**			
Formal (n=213)	Reference		
Informal (n=4,896)	2.3	1.5-3.7	<0.001
Not working (n=1,051)	2.3	1.4-3.7	0.001
**Covered by health insurance**			
No (n=1,106)	1.4	1.2-1.6	<0.001
Yes (n=5,054)	Reference	1.2-1.6	<0.001
**Place of ANC visit**			
Health post/dispensary (n=140)	1.4	0.9-2.4	0.119
Health center (n=5,649)	Reference		
District/provincial hospital (n=174)	1.1	0.7-1.6	0.538
Private or other (n=59)	1.2	0.6-2.3	0.475
Referral hospital (n=133)	0.5	0.2-0.8	0.01
**Total children ever born**			
Less than 3 (n=4,046)	Reference		
4-6 (n=1,657)	1.4	1.2-1.6	<0.001
7 and above (n=456)	1.5	1.2-2.1	0.001
**Wanted pregnancy**			
Yes (n=3,600)	Reference		
No (n=2,560)	1.7	1.5-2.0	<0.001

## Discussion

The purpose of this study was to determine the magnitude and factors associated with delayed ANC visits in Rwanda to facilitate decision making for the improvement prenatal care. From this study, the prevalence of delayed ANC visits was high (41%). However, this prevalence was lower than 62% reported in 2010 in Rwanda [14]. This may be due to increased sensitization of the community about reproductive health and engagement of community health workers that encourage pregnant women to seek care on time in Rwanda. This study revealed that women with no formal education, primary and secondary level education were more likely to have delayed ANC visits compared to those with higher education levels. The same findings were reported in Tanzania [22], Ethiopia [23], and Kenya [24], where it was indicated that women with higher education levels will attend their ANC on time. This may be due to the lack of knowledge about reproductive health including the significance of timely ANC visits which is acquired with the higher-level education.

The study also revealed that women who had more than four children were more likely to delay their ANC visits. This was supported by another study conducted in Rwanda [25] and Somaliland [26] where it was implied that women with a larger family size were more likely to delay their ANC visit. It could be that these women are too preoccupied with taking care of their children, believe that they have enough experience with pregnancy and see no point in having early ANC, or had poor previous ANC experiences.

Women who did not have health insurance were more likely to have delayed ANC visits. This was similar to other studies in sub-Saharan Africa [13], and Rwanda [14]. Even though ANC services are free of charge in Rwanda, there are some out pocket payments for services, such as laboratory investigations and consultation, when a mother needs them. These services are cheaper for people with insurance especially community-based insurance (mutuelle), which is the most common type used in Rwanda.

Women with informal jobs or unemployment were more likely to have delayed ANC visits. This is supported by another study conducted in Tanzania [22], where it was indicated that unemployed women were more likely to have late ANC. This may be because these women lack financial support to cater for their needs and thus are demoralized from attending ANC on time.

Surprisingly, age was not a risk factor for delayed ANC visit, contrary to other studies conducted in South Africa [27] and Kenya [24], where it was indicated that women below 20 years of age were more likely to have late ANC. These adolescent women may lack knowledge about the importance of ANC and consequences of delaying. In Rwanda, this could be due community health workers providing knowledge and encouraging all pregnant women to go have timely ANC irrespective of age.

## Conclusion

Early ANC services are crucial for the promotion of maternal and child health through early detection of pregnancy and childbirth related complications. The findings indicate that Rwanda still has a high prevalence of delayed ANC, with the risk factors being women´s occupation, women´s education, not having health insurance, having more than four children and unwanted pregnancy. Based on these findings, community-based family planning services should be made available to all women of childbearing age to prevent unwanted pregnancies, female education should be considered a priority, increase coverage of health insurance and community-based education about reproductive health to encourage early ANC.

**Limitations:** the DHS data did not include perception of pregnant women towards early ANC visit, experiences of women at ANC, quality of care at ANC. The survey was conducted on women who had a pregnancy in the five years preceding the survey thus recall bias was likely.

### What is known about this topic

- *According to a study done using data from Rwanda DHS 2010 delayed ANC was associated with having more than 6 children, having a problem with distance to health facility, and unwanted pregnancy*.

### What this study adds

- *Despite efforts to promote maternal health in Rwanda, delayed ANC prevalence is still an issue with a prevalence of 41% and was associated with low level of education of women, unwanted pregnancy, unemployment of women and not having health insurance*.
